# Aberrant Right and Left Gastric Veins as a Cause of Hepatic Pseudolesions: A Report of Three Cases

**DOI:** 10.7759/cureus.48455

**Published:** 2023-11-07

**Authors:** Alejandro J Quiroz Alfaro, Andrés Felipe Herrera Ortiz, Jose D Cardona Ortegón, Hannah Varney, Rodrigo Borrero León, David F Torres, Juliana Greiffenstein, Catalina A Dussan Tovar, Diego A Aguirre

**Affiliations:** 1 School of Medicine and Health Sciences, Universidad Colegio Mayor de Nuestra Señora del Rosario, Bogotá D.C, COL; 2 Department of Radiology, Fundación Santa Fe de Bogotá, Bogotá D.C, COL; 3 Department of Radiology, Universidad El Bosque, Bogotá D.C, COL; 4 School of Medicine, New York Institute of Technology College of Medicine at Arkansas State University, Jonesboro, USA; 5 School of Medicine and Health Sciences, Universidad Pontificia Bolivariana, Medellin, COL; 6 Department of Epidemiology, Universidad de La Sabana, Chía, COL

**Keywords:** aberrant liver anatomy, gastric cancer spread, hepatopetal flow, embryology, differential diagnoses, digital drawings, cross-sectional imaging, third inflow, hepatic pseudolesions, aberrant gastric veins

## Abstract

The complex structure of the liver and its elaborate hemodynamics can cause hepatic pseudolesions on contrast-enhanced imaging, making the interpretation of diagnostic liver imaging challenging. Aberrant gastric veins are rare; most of their epidemiology data comes from small single-center studies. While current literature suggests that pseudolesions originating from aberrant gastric veins mainly present as hyperdense defects, some cases can also show up as hypodense, as shown in these cases. Differences in flow rates between the portal and aberrant veins and the timing of the scans could explain this contradiction. Identifying aberrant gastric veins on cross-sectional imaging is crucial because they could be misdiagnosed as liver lesions, granting further unnecessary workups or invasive procedures. Aberrant gastric veins can also act as pathways for the spread of gastric cancer. This manuscript presents one aberrant right gastric vein and two aberrant left gastric veins causing hepatic pseudolesions.

## Introduction

The complex structure of the liver, its intricate blood supply with dual inflow, 25% from the hepatic arteries, and 75% from the portal vein, respectively, and the various pathological conditions that can impact liver function can make interpreting diagnostic liver imaging challenging [1]. Moreover, the complex hemodynamics of the liver may result in the appearance of hepatic pseudolesions (HPS) on contrast-enhanced imaging [2]. HPS are focal mass-like abnormalities mimicking actual liver lesions seen only on diagnostic imaging that can be misinterpreted, even by experienced radiologists, granting further unnecessary investigations, like biopsies [1,2].

During the early stages of embryonic development, anastomotic omental veins connect the venous plexus of the primitive foregut to the ductus venosus of Arantius [3]. Normal right and left gastric veins are formed due to the involution of these anastomotic veins; aberrant gastric veins can be formed secondary to the interruption of this physiological process [3].

Usually, gastric veins, which drain blood from the lesser curvature of the stomach, merge with the portal vein, making part of the physiological hepatopetal flow (blood flow toward the liver). Aberrant hepatopetal venous flow, also known as third inflow, may contribute to HPS; third inflow can be caused by an aberrant right gastric vein (ARGV) and, more rarely, by an aberrant left gastric vein (ALGV) [4].

We present three cases of patients with HPS secondary to aberrant gastric veins. Since not all the imaging features of aberrant gastric veins in cross-sectional imaging studies have been widely reported, we present these cases and encourage other authors to report similar cases.

## Case presentation

First case

**Figure 1:**
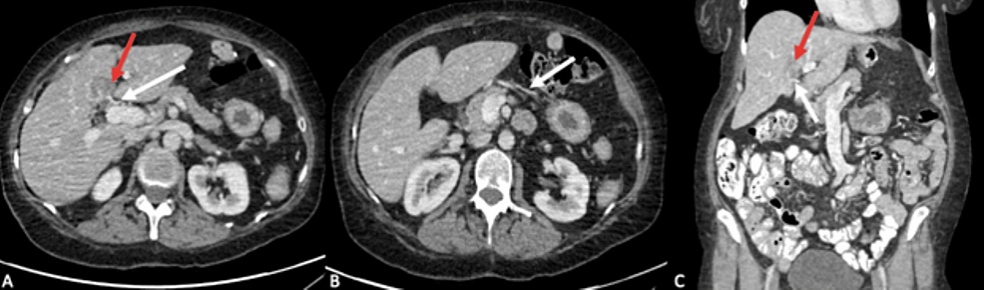
Contrast-enhanced abdominal CT Portal phase contrast-enhanced abdominal CT images (A, B, C) show an aberrant course of the right gastric vein (white arrows) draining into the hepatic segment IVb, consistent with an ARGV. A focal hypodense perfusion defect (red arrows) is visible in this location, which is traversed by blood vessels originating from the right gastric vein. ARGV: aberrant right gastric vein

**Figure 2:**
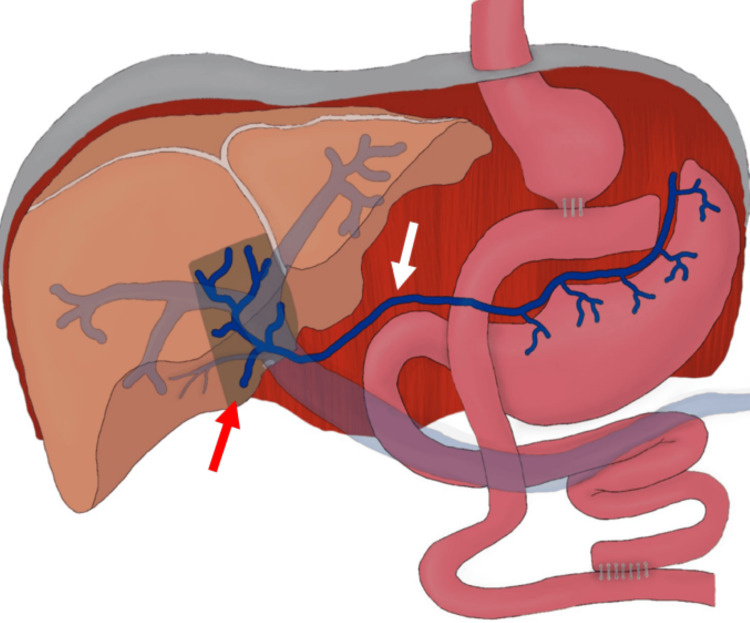
Digital drawing: first case Digital drawing illustrating the aberrant course of the right gastric vein (white arrow) draining into the hepatic segment IVb, consistent with an ARGV. A focal hypodense perfusion defect (red arrow) compatible with an HPS is also visible in this location. ARGV: aberrant right gastric vein; HPS: hepatic pseudolesions Source: elaborated by the authors

An asymptomatic, 62-year-old woman with a history of a partial gastrectomy with gastrojejunal anastomosis and cholecystectomy underwent an abdominal contrast-enhanced computed tomography (CT) to assess a renal angiomyolipoma identified incidentally on a routine abdominal ultrasound. The CT showed an aberrant drainage pattern of the right gastric vein into the liver, causing a hypodense HPS localized at the hepatic segment IVb (Figures 1, 2; Video 1). 

**Video 1:** Portal phase contrast-enhanced abdominal CT CT image showing an aberrant course of the right gastric vein (arrows) draining into the hepatic segment IVb, consistent with an ARGV. ARGV: aberrant right gastric vein

Since the patient remained asymptomatic, no further intervention was necessary.

Second case

**Figure 3:**
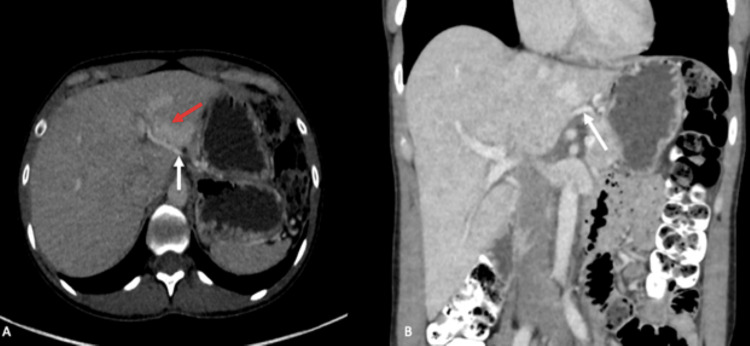
Contrast-enhanced abdominal CT Portal phase contrast-enhanced abdominal CT images (A, B) showing an aberrant course of the left gastric vein (white arrows) draining into the posterior margin of the hepatic segment III, consistent with an ALGV. A focal hyperdense perfusion defect (red arrow) perfused by blood vessels originating from the left gastric vein is also visible in hepatic segments II and III, compatible with an HPS. ALGV: aberrant left gastric vein; HPS: hepatic pseudolesion

**Figure 4:**
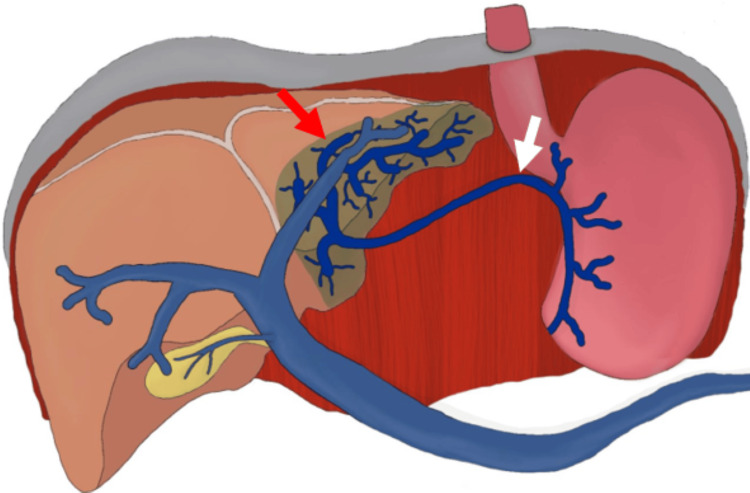
Digital drawing: second case Digital drawing illustrating the aberrant course of the left gastric vein (white arrow) draining into the hepatic segment III, consistent with an ALGV. A focal hyperdense perfusion defect affecting hepatic segments II and III (red arrow) compatible with an HPS is also visible in this location. ALGV: aberrant left gastric vein; HPS: hepatic pseudolesion Source: elaborated by the authors.

A 46-year-old woman with a medical history of irritable bowel syndrome (IBS) arrived at the emergency department with dull abdominal pain lasting for two months. During the workup, the patient underwent a contrast-enhanced abdominal CT, which incidentally revealed an ALGV, causing a HPS localized at the hepatic segments II and III (Figures 3, 4; Video 2).

**Video 2:** Portal phase contrast-enhanced abdominal CT CT showing an aberrant course of the left gastric vein (arrows) draining into the posterior margin of the hepatic segment III, consistent with an ALGV. ALGV: aberrant left gastric vein

Since the ALGV was an incidental finding, the patient was conservatively treated.

Third case

**Figure 5:**
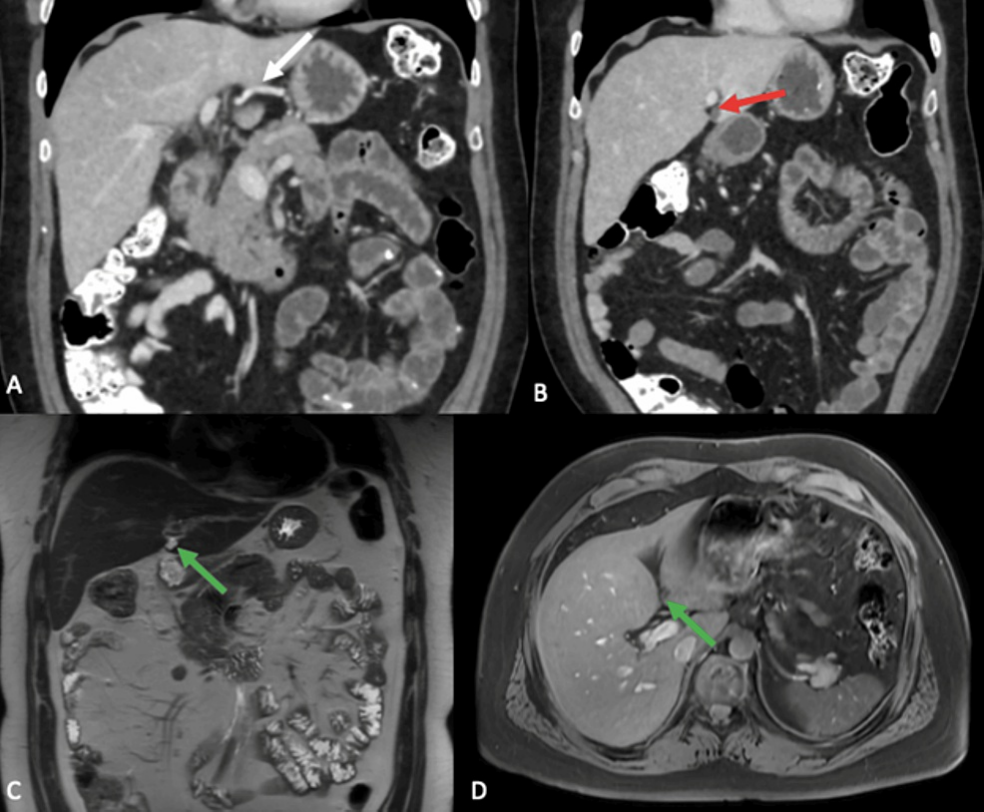
Contrast-enhanced cross-sectional imaging Portal phase contrast-enhanced abdominal CT (A, B) showing an aberrant course of the left gastric vein (white arrow) draining into the posterior margin of the hepatic segment III, consistent with an ALGV. A focal hypodense perfusion defect traversed by blood vessels in its posterior aspect, compatible with an HPS is also found in hepatic segment III (red arrow). A contrast-enhanced MRI (C, D) showed the same perfusion defect (green arrows) compatible with an HPS, as hyperintense on T2 and hypointense on T1. ALGV: aberrant left gastric vein; HPS: hepatic pseudolesion

A 50-year-old man with a history of abdominal pain lasting one week underwent a contrast-enhanced abdominal CT, which incidentally revealed an ALGV, causing a small HPS localized at hepatic segment III (Figure 5; Video 3).

**Video 3:** Portal phase contrast-enhanced abdominal CT Abdominal CT showing an aberrant course of the left gastric vein (arrows) draining into the posterior margin of the hepatic segment III, consistent with an ALGV. ALGV: aberrant left gastric vein

The patient's abdominal pain was attributed to IBS after ruling out alternative diagnoses; therefore, he was medically managed and discharged after resolution.

## Discussion

Aberrant gastric veins are considered an exceedingly rare finding. Most information regarding their epidemiology comes from small single-center studies. The incidence of ALGV ranges from 0.8%-4%, whereas ARGV is 1.5%-49%, as reported by Kobayashi [2]. In our experience, the three cases reported here are the only ones we have encountered incidentally on cross-sectional imaging studies during our clinical practice.

According to Muñoz and Fraum, the ALGV typically drains into the posterior margins of hepatic segments II and III [5]. At the same time, the ARGV is mentioned to drain more often into the posterior margin of segment IV, consistent with the findings documented in all our cases. It is essential to know that the HPS associated with an aberrant gastric vein typically affects the exact segment the aberrant vein drains into, as shown in our case series.

Elsayes et al. described that the HPS associated with either aberrant gastric vein results in an area of relative hyperenhancement at contrast-enhanced CT and MRI [6]; nonetheless, we have found that although the HPS in our second case matches the description, the HPS of our first and third cases do not, manifesting as a hypodense pseudolesion. This could be explained by the fact that the flow rate through the aberrant gastric vein is typically lower than that of the portal vein. Consequently, during a contrast-enhanced CT or MRI scan of the liver, the HPS may not enhance equally as the rest of the liver, resulting in a variable hypodense or hyperdense appearance.

We consider that the hypodense or hyperdense appearance of the HPS in imaging studies depends on the timing of the scan rather than on the intrinsic characteristics of the lesion. Considering these factors carefully when interpreting imaging studies is critical to avoid misdiagnosing and ensure appropriate clinical management.

Some authors have described additional clinical significance for aberrant gastric veins; for example, these could act as an alternative route for venous drainage in hypertensive gastropathy in cirrhotic patients or as a direct metastatic pathway for gastric cancer to the left side of the liver [3,5]. Moreover, it is critical to know this anatomical variation in patients planning for gastric or hepatic surgery since this could be a source of accidental hemorrhage, potentially increasing the time of surgery and morbidity [3,5]. More cases like ours should be reported to increase the available evidence on imaging features of aberrant gastric veins in cross-sectional imaging studies.

## Conclusions

Aberrant gastric veins are a rare but relevant finding in cross-sectional imaging, potentially leading to additional imaging studies or biopsies. The density of the HPS caused by aberrant gastric drainage can be variable, ranging from hyperdense to hypodense, depending on the timing of the scan rather than the lesion's intrinsic characteristics.

Encouraging authors to publish more cases like ours is crucial to increasing the available evidence on aberrant gastric vein imaging features in cross-sectional imaging studies.
